# MicroRNAs in the Mouse Developing Retina

**DOI:** 10.3390/ijms24032992

**Published:** 2023-02-03

**Authors:** Jorge Navarro-Calvo, Gema Esquiva, Violeta Gómez-Vicente, Luis M. Valor

**Affiliations:** 1 Unidad de Investigación, Hospital General Universitario Dr. Balmis, ISABIAL, 03010 Alicante, Spain; 2 Department of Optics, Pharmacology and Anatomy, University of Alicante, 03690 Alicante, Spain

**Keywords:** retina, mouse, Dicer, miR-183/96/182, miR-204/211, miR-124, miR-9, let-7, transcriptomics

## Abstract

The retina is among the highest organized tissues of the central nervous system. To achieve such organization, a finely tuned regulation of developmental processes is required to form the retinal layers that contain the specialized neurons and supporting glial cells to allow precise phototransduction. MicroRNAs are a class of small RNAs with undoubtful roles in fundamental biological processes, including neurodevelopment of the brain and the retina. This review provides a short overview of the most important findings regarding microRNAs in the regulation of retinal development, from the developmental-dependent rearrangement of the microRNA expression program to the key roles of particular microRNAs in the differentiation and maintenance of retinal cell subtypes.

## 1. Biogenesis of microRNAs

In the last two decades, non-coding RNAs (ncRNAs) have emerged as crucial regulators in cell biology. These transcripts are not translated into proteins but, instead, remain as processed RNA molecules with regulatory activities that can be classified into long and short ncRNAs according to their size. Long non-coding RNAs (lncRNAs) are heterogeneous from the point of view of their biogenesis and genomic origin, but all of them have in common an average length above 200 nucleotides and lack canonical coding sequences. Biological functions of lncRNAs include the regulation of chromatin compaction, the suppression of gene expression by interfering with the transcription machinery, and the interaction with proteins as scaffolds, among others (reviewed in [1]). The most investigated classes of short non-coding RNAs (sncRNAs) are piwi-interacting RNAs (piRNAs), endogenous small interfering RNAs (siRNAs), and microRNAs (miRNAs). Unlike siRNAs and miRNAs, which are extensively expressed in most mammalian tissues, piRNAs are expressed in the germline where they contribute to the maintenance of genomic stability by targeting and repressing the expression of transposable and repetitive elements [1]. Endogenous siRNAs molecules derive from long double-stranded RNA precursors that undergo cleavage in the cytoplasm by the endoribonuclease Dicer, giving way to smaller (21–23-nucleotide-long) RNA molecules. Each resulting siRNA is able to inactivate the expression of one specific target gene, as one of its strands binds to the corresponding messenger RNA (mRNA) through fully complementary base pairing. Similarly, miRNAs are key post-transcriptional regulators of gene expression [2], and consist of non-coding RNAs of 18–22 nucleotides that bind to partially complementary regions of target mRNAs to repress their translation or promote their degradation. These molecules derive from primary RNA transcripts (pri-miRNA) generated by RNA polymerase II [3]. In the canonical miRNA biogenesis pathway, pri-miRNAs self-anneal to form one or a few stem-loop structures that are further processed, in the cell nucleus, to 65–70-nucleotide-long sequences known as precursor miRNAs (pre-miRNAs). This processing involves cleavage by the microprocessor complex, a heterotrimeric complex with endoribonuclease activity, consisting of one Drosha and two DGCR8 molecules [4]. In a non-canonical biogenesis pathway, pre-miRNAs also result from Drosha and DGCR8-independent splicing [5]. The resulting pre-miRNAs are then exported to the cytoplasm by the nucleocytoplasmic transport factor Exportin 5 [6], where the endoribonuclease Dicer cleaves them into approximately 22-nucleotide-long double-stranded miRNA molecules [7]. In the last processing step, the miRNA duplex is assembled with the Argonaute 2 protein to form a ribonucleoprotein complex known as RNA-induced silencing complex (RISC). During RISC assembly, one of the strands in the miRNA duplex is eliminated to render a single-stranded mature miRNA, which is guided to its specific mRNA target through base pairing [8]. Upon binding of the seed sequence (usually the first 2–8 nucleotides located at the miRNA 5′-end) to the mRNA (generally but not restricted to the 3′UTR), miRNAs either induce the degradation of mRNA transcripts or reduce their translational efficacy. Because a single miRNA can have many mRNA targets as pairing is based on short sequences in which certain mismatches are allowed, it turns into a potent regulator of gene expression.

## 2. Brief Overview of the Retinal Development

The mature vertebrate retina is a layered nervous tissue composed of six major neuronal types including primary sensory cells (rod and cone photoreceptors), interneurons (horizontal, bipolar, and amacrine cells), and output neurons (retinal ganglion cells, RGCs). These neurons form functionally and morphologically distinct circuits that operate coordinately to produce a complex visual output. All vertebrate retinas share a fundamental architecture consisting of the above-mentioned neuronal types distributed in three different cell body layers separated by two synaptic or plexiform layers. In addition, Müller glia contributes to the maintenance of tissue homeostasis by providing structural and metabolic support to retinal neurons [9]. Despite a common structural-functional retina among vertebrates, variations to the basic design can be found across species. The most distinctive one is in the composition of retinal photoreceptors and their circuitry. In rodents, rods are the most abundant retinal cells (72%), followed by bipolar and amacrine cells (10% and 8%, respectively). Cones, RGCs, and Müller glia comprise 2–3% of retinal cells each, while horizontal cells represent only 0.3% [10]. Moreover, three types of cone photoreceptors coexist in the mouse retina: the majority (54%) co-express middle- and short-wavelength opsins, whereas 37% and 10% express exclusively middle- or short-wavelength opsins, respectively [11].

**Figure 1:**
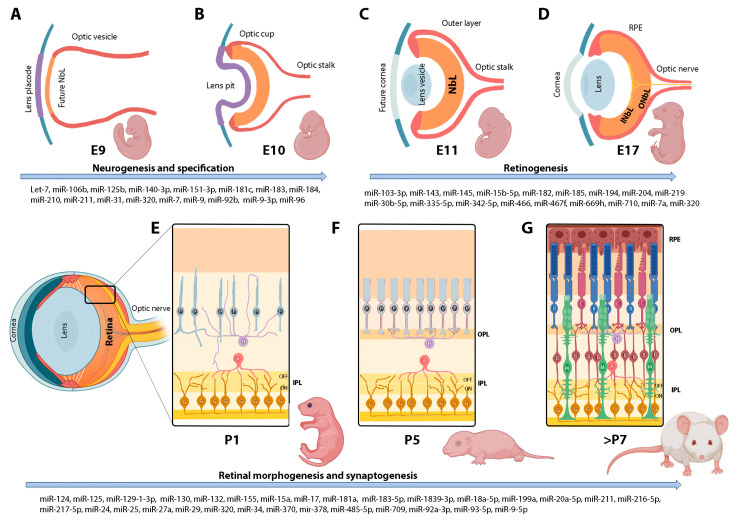
Schematic summary of the developing mouse retina and main miRNAs involved. (**A**–**D**): Schematic showing the various stages of eye development, from optic vesicle formation until the specification of the outer (ONbL) and inner neuroblastic layer (INbL). (**E**–**G**): Schematic showing the neural stratification and the sequence of circuit assembly in the mouse retina. E = embryonic day; P = postnatal day. IPL = inner plexiform layer; OPL = outer plexiform layer; RPE = retinal pigment epithelium. G = retinal ganglion cells; A = amacrine cells; H = horizontal cells; B = bipolar cells; P = Photoreceptors; C = cones; R = rods; M = Müller glia. Figure created with BioRender (https://biorender.com/, URL accessed on 20 January 2023).

The mouse retina derives from regions adjacent to the ventral diencephalon. On embryonic day (E) 10, the cavities produced by invagination of the optic vesicles hollow out, modeling a double-layered optic cup. The inner layer of the optic cup, known as the neuroblastic layer (NbL), is a proliferative zone made up of retinal progenitor cells (RPCs) that, around E17, differentiates into an inner neuroblastic layer (INbL) and an outer neuroblastic layer (ONbL). The INbL will give rise to the retinal ganglion cell layer, while the ONbL will form the inner nuclear layer (INL), containing the somas of horizontal, bipolar, and amacrine neurons, and the outer nuclear layer (ONL), comprising the nuclei of cone and rod photoreceptors [12]. All these retinal neurons and glial cells are generated in an orchestrated order thanks to the confluence of regulatory mechanisms for cell proliferation and specification [13]. Retinal morphogenesis also includes additional processes such as retinal patterning, synaptogenesis, and apoptosis, which, together, ensure a complete functional retina (Figure 1).

### 2.1. Regulation of Retinal Neurogenesis and Specification

During neurogenesis, intrinsic and extrinsic cues regulate the generation of specific retinal cell types and subtypes in the appropriate numbers. Some of the intrinsic factors that play a relevant role in cell fate specification are Visual System Homeobox 2 (VSX2 aka CHX10), Paired Box 6 (PAX6), Retina and Anterior Neural Fold Homeobox (RAX), and Sine Oculis Homeobox Homolog 3 (SIX3). These four factors, together with Sonic Hedgehog Signaling Molecule (SHH), are involved in retinal progenitor proliferation as well. Other transcription factors that regulate specification are Neural retinal leucine zipper (NRL) and Nuclear receptor class 2, subfamily e, member 3 (NR2E3), expressed selectively in developing rods, and POU Domain, Class 4, Transcription Factor 2 (POU4F2 aka BRN3B), expressed in developing and mature RGCs (reviewed in [9,12]). Among the extrinsic factors that modulate retinal neurogenesis and cell differentiation are Shh, fibroblast growth factors, Wnts, bone morphogenetic proteins (BMPs) and the related growth differentiation factors (GDFs), Notch, and retinoic acid [9].

### 2.2. Regulation of the Morphology and Connectivity of Retinal Neurons

Dendritic arborization of retinal neurons such as amacrine and RGCs is modeled by isoneural and heteroneural repulsion, terms that refer to the minimal branch overlapping between dendrites of the same cell or neighbor cells, respectively (reviewed in [14]). Using knock-out (KO) mouse models, several “self-avoidance” molecules have been identified in the retina, including the protein Down-syndrome cell adhesion molecule (DSCAM) [15], the semaphorins and their receptors plexins [16], and the protocadherins [17]. Therefore, arborization patterns of the aforementioned neurons, as well as arborization-dependent connectivity among neighbors of the same type, are regulated by the coordinated expression of avoidance proteins [14]. Likewise, the interactions of retinal neurons with their presynaptic partners also influence morphogenesis. This is the case of bipolar and horizontal cells, whose number of dendritic branch terminals and the regularity of their spacing depend on the density of their presynaptic cone photoreceptors [14,18].

In the outer plexiform layer (OPL) of the retina, photoreceptors establish contact with postsynaptic neurons, bipolar and horizontal cells, in a specialized synapse called a triad, which consists of an invagination of the photoreceptor axon terminal (a spherule in the case of rods and a pedicle in cones) that contains two lateral horizontal dendritic tips surrounding one central bipolar process. These types of invaginating synapses contain synaptic ribbons that release glutamate continuously in the darkness. In addition, bipolar dendrites form non-invaginating basal synapses with photoreceptor terminals [19], and other synaptic contacts occur outside invaginating synapses via gap junctions between horizontal–bipolar cell processes, and horizontal–horizontal cell processes. During the first two weeks of postnatal development, synapse assembly in the OPL follows a sequential pattern: first, a photoreceptor terminal contacts with a single horizontal cell process and, shortly after, a dyad synapse is formed when another horizontal cell is recruited into the synaptic complex. Then, the horizontal processes start invaginating into the photoreceptor terminal and the triad arises when a bipolar cell dendrite inserts in the middle of the two horizontal tips. Synaptogenesis is paralleled by the expression of presynaptic proteins at the photoreceptor ribbon synapse such as RIBEYE (aka CTBP2), Bassoon, the postsynaptic density protein PSD-95, or the vesicular glutamate transporter 1 (VGlut1), and postsynaptic proteins such as the metabotropic glutamate receptor 6 (mGluR6) or the ionotropic glutamate receptors Gluk5, GluK2/3, GluA1, GluA2/3, and GluA4 [14]. A role for adhesion proteins has been established during synapse assembly. Some examples are the rod-terminal-specific adhesion protein SynCAM1, important for the maturation of the rod ribbon synapse [20]; the anchoring protein dystroglycan, required for invagination of bipolar processes within the rod terminal, and its binding partner, the extracellular matrix protein pikachurin [21]; the synaptic adhesion protein netrin-G ligand2 (NGL-2 aka LRRC4), localized on horizontal cell processes [22]. Besides molecular cues, neuronal activity also contributes to the assembly and maintenance of functional synapses.

Likewise, in the course of retinal development, two types of synapses are established in the inner plexiform layer (IPL) of the retina: the excitatory and the inhibitory synapses. Excitatory synapses are ribbon synapses established between the axons of bipolar cells and the dendrites of their postsynaptic neurons, which are two amacrine cells in the case of rod bipolar cell synapses, or one amacrine and one RGC in the case of cone bipolar cell synapses [14]. Bipolar ribbon synapses differ from those of photoreceptors in that the former do not express the presynaptic protein Bassoon [23] and in that PSD95 is found postsynaptically, instead of presynaptically, in RGCs [24]. Ionotropic glutamate receptors mediate amacrine and RGCs responses on the postsynaptic side of the ribbon synapse; therefore, Gluk5, GluK2/3, GluA1, GluA2/3, and GluA4 increase their protein expression as the retina matures (from P0 to P30) [14]. Inhibitory synapses, in turn, modulate the flow of information through the IPL. These are established between diverse amacrine cell subtypes and bipolar cells, RGCs, or even other amacrine cells, and are mediated by glycine or γ-aminobutyric acid (GABA) neurotransmitters. Synaptogenesis in the IPL of the mouse retina follows a precise order in which a rise in amacrine–amacrine (inhibitory) synapses from P0 to P10 precedes the increase in ribbon (excitatory) synapses from P11 to P15. Finally, both excitatory and inhibitory synapses experience a significant reduction in their numbers that takes place around eye opening, approximately at P15 [25].

As above-stated, RGCs constitute the output neurons of the retina. Therefore, their axons travel through the retinal nerve fiber layer and gather at the optic nerve head, where they abandon the eye and progress toward the diencephalon ventral midline. Then, they must face the decision of crossing or avoiding the midline at the optic chiasm. In animal species with poor binocular vision, i.e., none or little overlap of the right and left visual fields, all RGCs axons cross the midline and project to targets on the contralateral brain hemisphere. On the contrary, in species characterized by proper binocular vision, as is the case of the mouse, the RGCs axons project ipsilaterally, as well as contralaterally [26,27]. At E12.5, RGCs that emerge from the dorso-central mouse retina approach the optic chiasm and turn ipsilaterally before reaching it. The peak phase of midline guidance takes place between E14 and E17, when most axons reach the optic chiasm and are guided either contralaterally or ipsilaterally. Finally, late-born RGCs project mainly to the contralateral side from E17 to P0 [27,28]. RGC axons continue growing from the chiasm into the optic tracts to reach their targets, with the superior colliculus and the lateral geniculate nucleus being the ones that receive the majority of retinal inputs. There, ipsilateral and contralateral RGCs axons rearrange in a topographic manner, shifting from initially disorganized maps, where axons overlap and extend beyond their final topographic region, to precisely organized retinotopic maps, where neighboring neurons in the retina project to neighboring neurons in the lateral geniculate nucleus. Retinotopic map formation in the mouse occurs during the first postnatal week and involves a refinement process that courses with RGCs loss and pruning of their arbors driven by neuronal-activity-dependent and -independent mechanisms [27,29].

### 2.3. Regulation of Retinal Apoptosis

While a large number of cells are generated during mouse retinal neurogenesis, those that are transiently present or do not migrate properly during development are eliminated by apoptosis. A second wave of retinal cell death takes place during synaptogenesis. Differentiated retinal neurons begin to establish synaptic contacts with their targets, which supply them with critical trophic factors. Failure to obtain trophic support triggers neuronal apoptosis [30]. As a result, the retina becomes a functional, paired network between neurons and their target cells. In the fully developed retina, however, the emphasis must shift toward survival; therefore, regulatory mechanisms come into play to prevent inappropriate cell death. Thus, retinal neurons progressively downregulate their apoptotic machinery as they mature [31,32], making use of epigenetic mechanisms at the transcriptional level [33,34] or miRNA at the post-transcriptional and translational levels [35].

## 3. Retinal microRNAs in Development

### 3.1. Dissecting the Roles of microRNAs during Retinal Development

To elucidate the functional roles of miRNAs during retinal development, either candidate-oriented or global unbiased studies have been conducted to provide a detailed description of their temporal expression to clarify the regulatory network of target gene effectors. A more sophisticated approach is based on the characterization of genetically manipulated mice, considering that at least one third of the retinal miRNAs are expressed in both mouse and human retinas [36]. Therefore, mouse models are suitable model systems for the study of the regulatory pathways of retinal miRNA, where experimental disruption can cause development-dependent visual impairments that may be reminiscent of eye diseases for which no treatment is currently available [37]. Table 1 summarizes the most relevant miRNAs found in research focused on retinal development and cell differentiation.

**Figure 2:**
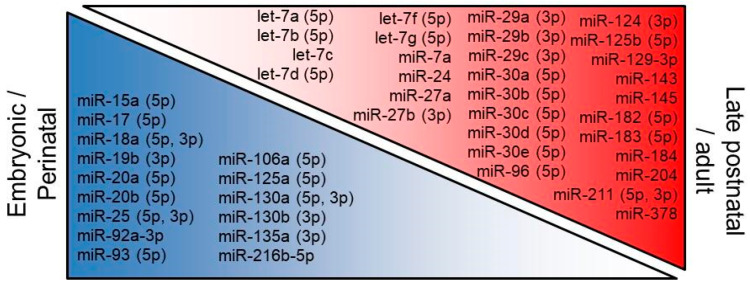
Summary of the most consistent changing miRNAs in miRNome research (blue, downregulated; red, upregulated) that at least have been retrieved in two of the following studies: [39,41,42,43,46,47,49,51,55,56,58,61,62,63]. The arm (5p or 3p) is indicated when specifically reported.

As referred in Figure 1 and Table 1, the study of the miRNA transcriptome (miRNome) in the retina has revealed that several miRNAs are developmentally regulated during embryonic and early postnatal stages, suggesting that these distinctive expression patterns may influence the activities of downstream targets that specify retinal cell types. Although some miRNAs exhibit complex waves of expression, as in the examples of miR-143 or miR-9, it is possible to describe a simplified pattern of down- and up-regulation as the maturation of the retina progresses (Figure 2).

In fact, most of these tightly regulated miRNAs actually play substantial roles during development. In the following sections, we provide some relevant examples.

#### 3.1.1. The miR-182/96/183 Cluster

For instance, the miR-182/96/183 cluster is indispensable for the development and postmitotic maintenance of photoreceptor outer segments in the retina [37]. The miR-183/96/182 cluster encompasses approximately 3.8 kb in chromosome 6 (mouse) or 7 (human) and has been originated by gene duplication [64]; therefore, miR-183, miR-96, and miR-182 have very similar seed sequences [65], although just a single base difference can change the binding properties to the mRNA target sequence [66,67]. The three miRNAs are coexpressed from a single transcript [42], resulting in a pri-miR-183/96/182 that is highly expressed in early developmental stages. However, the appearance of the mature miRNA pool, prominently in the INL and mature photoreceptors [42], is delayed due to RNA helicase activity [68]. Disruption of the whole cluster leads to early and progressive synaptic disruption of photoreceptors [69]. However, a preliminary report indicated that the conventional miR-182 KO failed to produce a phenotype [70], suggesting that this miRNA was dispensable or at least its ablation was compensated by the other members, but a closer inspection determined retinal alterations at the photoreceptor level [58]. Using CRISPR-Cas technology, it has been possible to precisely remove specific sequences from the adjacent sequences for miR-183 and miR-96, leading to similar although not exact results in the differentiation and maintenance of photoreceptor functions [52,59]. Among the specific roles of the cluster are the regulation of Pax6-dependent retinal morphogenesis [71], proper adhesion of photoreceptors and Müller glial cells [68], and formation of the synaptic connections between photoreceptors and retinal postsynaptic cells [69].

#### 3.1.2. The miR-204/211 Family

Members of the miR-204/211 family show an evolutionarily conserved expression across several ocular regions such as the RPE, neural retina, ciliary body, and lens [72]. As miR-211 appeared in mammals from a gene duplication event from miR-204 [72], both miRNAs only show one nucleotide difference between mature sequences, with an identical seed sequence that may target similar sets of genes [73]. MiR-204 is the most studied member of the family and regulates key genes during embryogenesis and optic nerve formation. It participates in axon guidance and promotes cell polarity and retinal maturation. It is also involved in different processes such as metabolism, oxidative stress, nerve protection, cell junctions, and neurogenesis [73]. MiR-204, even at low levels of expression, is still able to play an important role in the maintenance of retinal photoreceptor cells [72]. Similarly, the removal of miR-211 in the mouse retina compromises cone photoreceptor function and survival [60].

#### 3.1.3. miR-124

Reducing the miR-124 levels during development reduces opsin expression and leads to cone photoreceptor death [53]. The miR-124 family represents the most abundant miRNAs (25% of the total miRNAs) expressed in the brain [74], whose dysregulation is linked to neurodegenerative disorders such as Alzheimer’s disease (AD), Parkinson’s disease (PD), and Age-related Macular Degeneration (AMD) [75], a condition characterized by loss of the central vision as a result of RPE and photoreceptor cell death and choroidal neovascularization, whose cause is still elusive [76].

#### 3.1.4. miR-9

miR-9 is highly conserved between species and is mainly expressed in the CNS. This miRNA modulates neuronal proliferation and differentiation in the brain and spinal cord in vertebrates [77], and many miR-9 functions are related to CNS development such as neural stem cell proliferation and differentiation, reaching elevated levels in adulthood [74,78]. Specifically, miR-9 is expressed in retinal progenitors in vivo and its expression increases between early embryonic stages and the postnatal period of neurogenesis [39]. In cultured retinal progenitors derived from P1 C57BL/6 mice, it was shown that miR-9 targeted the orphan nuclear receptor TLX, which promotes proliferation, shifting the balance toward neuronal and glial differentiation [79]. When overexpressed in retinal progenitors in vivo, it suppressed retinal progenitor differentiation into glial cells, promoting their differentiation into neurons [45]. In addition, miR-9 has been involved in diverse neurodegenerative conditions such as AD [80], amyotrophic lateral sclerosis (ALS) [81], and Huntington’s disease (HD) [82].

#### 3.1.5. The Let-7 Family

Let-7 (lethal-7) is the first known miRNA in animals, first identified in *C. elegans*, whose sequence and function are conserved across different species . The let-7 family members consist of let-7a through let-7k, miR-98, and miR-202 [83,84] and regulate multiple processes such as apoptosis, immune system modulation, axon guidance, regeneration, sleep, blood–brain-barrier maintenance, and cellular senescence [85,86,87,88,89,90,91,92]. Let-7 is expressed in both embryonic and adult brains with roles in cell development and maturation. Whereas let-7a has been reported to be involved in neural cell differentiation [93], let-7b reduces self-renewal of aged neural stem cells and shortens the cell cycle of neural progenitors [94]. Like miR-9, let-7a is expressed in retinal progenitors at early embryonic stages (E10) and its levels increase with age until the early postnatal period (P3). Together, these miRNAs are required for the change in competence of the retinal progenitors and, when their expression is suppressed, the retinas display higher numbers of photoreceptors, horizontal cells, and retinal ganglion cells [39].

### 3.2. Developmental Regulation of Retinal microRNAs

How are these miRNAs regulated during retinal development? As already commented in this review, Dicer is responsible for miRNA biogenesis, although the study of genetically manipulated mice indicates that this biogenesis is complex. In contrast to conventional KO that shows embryonic lethality [95], the impact of Dicer ablation in the brains of conditional KO has been more subtle than expected due to, for example, mosaicism of the mutation, high stability of existing miRNAs, residual amounts of Dicer, and Dicer-independent miRNA processing [96,97,98]. In the case of the retina, *Dicer1* KO in mouse RPC (thanks to the use of a Cre recombinase targeted by *Vsx2*/*Chx10* regulatory sequences) has no obvious phenotype caused by developmental impairments but is characterized for a retinal degeneration at the second postnatal week [98]. Nonetheless, a posterior mutant in which Dicer was removed from Pax6-expressing cells demonstrated that this key component of miRNA maturation has a relevant role in RPC competence and retinal neuron differentiation. This was postnatally reflected by the presence of ectopic retinal ganglion cells and the lack of other types of mature neurons [38], highlighting the importance of miRNAs in the correct development of the retina. Even long after rod specification, miRNAs seem to play an essential role in supporting photoreceptor survival, as the disruption of miRNA processing due to the loss of Dicer at P28 led to photoreceptor outer segment disorganization followed by retinal degeneration in 8- and 14-week-old mice, respectively [99]. In another paradigm, Dicer was depleted in cones (thanks to the use of a Cre recombinase targeted by *Chrnb4* regulatory sequences) and, though cKO mice displayed normal retinogenesis, with all cone precursor cells being born by E17, cone inner and outer segments were impaired or absent by P21. Loss of Dicer also affected the maturation and/or survival of ganglion and horizontal cells, in addition to cone photoreceptors. Cone degeneration progressed by 3.5 and 6 months and was accompanied by impaired cone function [100]. MiRNAs seem to be equally indispensable for RGCs axon guidance, as Dicer loss led to severe axon pathfinding defects at the optic chiasm [101].

More specifically, the nuclear hormone receptors NR2E3 and RORA (Retinoic-acid-receptor-related orphan receptor alpha), as members of the ligand-dependent steroid hormone receptors that function as transcription factors, regulate the generation of cone and rod cells and have relevant implications in AMD pathogenesis, with confirmed directed binding to miRNA-containing loci [102,103,104,105,106]. Interestingly, the transcription factor associated with microphthalmia MITF (Melanocyte-induced transcription factor) regulates the RPE differentiation through modulating the expression of miR-204/211 [107]. NRL is a transcriptional activator of rod-specific genes that has been tightly involved with the most prevalent cause of blindness in adult population of genetic origin, the retinitis pigmentosa [108]. Ablation of NRL in mice leads to the transcriptional dysregulation of miRNAs, including the upregulation of miR-184, miR-204, and miR-211 [49]. It is also a regulator of the miR-143/145 cluster as part of a feedback mechanism [55]. PTF1A, first discovered as a pancreas-associated transcription factor, regulates amacrine and horizontal cell fate [109], and its removal provokes the downregulation of miR-216a/b that are necessary for amacrine cell formation [61]. The retinal noncoding RNA 4 (Rncr4) is expressed in the opposite direction of the pri-miR-183/96/182 in photoreceptors undergoing maturation and modulates the activity of the DEAD-box RNA helicase/ATPase DDX3X to inhibit the processing of the pri-miR-183/96/182 in early postnatal photoreceptor cells, causing the thinning of the photoreceptor layer and INL [68]. All these examples indicate that a complex network of transcription factors, cofactors, and miRNAs are orchestrated in order to refine retinal development. However, our current knowledge regarding this network is too limited, and we still need to elucidate how most of the intrinsic and extrinsic factors (see Section 2.1) can regulate the expression of miRNAs to exert their multiple roles in cell fate determination and maintenance.

## 4. Involvement of microRNAs in Retinal Pathologies

Due to the physiological role of miRNAs in the regulation of metabolism, inflammation, angiogenesis, and retinal development, an imbalance in their expression or activity can lead to different retinal pathologies. Up until now, a single congenital human condition has been associated with retinal miRNAs: the nonsyndromic retinal dystrophy and iris coloboma with or without congenital cataracts (RDICC, OMIM #616722) in which retinal atrophy mainly affects RPE, reduces retinal vasculature, attenuates light responses, and other clinical features, is linked to a point dominant mutation (n.37C > T) in the seed sequence of miR-204 [110]. MiRNAs are also involved in retinopathy of prematurity (ROP), a vascular disease characterized by abnormal vessel development in the retina of premature babies that can lead to blindness. In this pathology, miR-18a-5p and miR-145 were found to be upregulated, disrupting angiogenesis in the retina, while the miR-34a, miR-96, miR-150, miR-181a-5p, and miR-182-5p were downregulated, inhibiting retinal neovascularization and vessel loss [111].

Beyond these examples, little is known regarding the involvement of miRNAs in retinal developmental disorders. However, developmental and adult disorders affecting the retina may share pathological mechanisms, and lessons can be learnt from the study of these latter conditions that can be applied to neovascularization, neuronal apoptosis, and cell identity during development. For instance, the ROP-associated miR-150 is also downregulated in the neural retina of patients with diabetic retinopathy (an ocular complication of diabetes affecting blood vessels), promoting inflammation and apoptosis of photoreceptors and contributing to the microvascular degeneration [112]. MiRNA dysregulation has been described in cancer as well. In retinoblastoma, the most common ocular pediatric malignancy, miR-494 and miR-9, were up- and downregulated in retinoblastoma, respectively; although oppositely deregulated, these miRNAs share the same targets as evidenced in the analysis of tumoral-originated exosomes [113]. Another pathology is glaucoma, an important cause of irreversible blindness caused by nerve optic injuries, for which nearly 200 miRNAs have been described among regulatory molecules as candidates for having roles in the pathogenesis [114]. In addition, miRNAs are also an important regulator of many age-related diseases such as the AMD, which suggests that miRNAs might influence lipid metabolism during lesion development [115]. Indeed, the exposure to oxidative stress determines the altered expression of miRNAs that might be implicated in the etiopathogenesis and progression of retinitis pigmentosa [116]. Finally, miRNAs have also been implicated in ocular autoimmune disorders, such as autoimmune uveitis, Grave’s ophthalmology, and Sjögren’s syndrome dry eye. Due to the important roles of miRNAs in regulating inflammation and the immune response, miRNAs can be potentially therapeutic targets for autoimmune-mediated eye disease [117]. Establishing how miRNAs contribute to these ocular pathologies and the finding of new regulative functions of miRNAs can potentially be valuable diagnostic or prognostic biomarkers and will help to open new treatments for these diseases.

## 5. Perspectives

Retina development is a highly orchestrated process for a highly organized tissue. Transcriptional and epigenetic regulation of multiple miRNAs during retinal cell migration and differentiation extend their impact over the activity of hundreds of genes as a single miRNA can potentially control several target genes in a simultaneous manner to influence the function of multiple pathways [118,119]. Computational predictions envisage a complex landscape of miRNA–target gene interactions and pathways regulation, but we still need functional validations that take into consideration the temporal and spatial events, including intercellular interactions, during retinal development.

Dissection of the key miRNAs during this process is challenging due to the redundancy of seed sequences and target genes that otherwise ensure a correct maturation of retinal structures for precise functioning. Although each member of a miRNA family can exert subtle specific roles that cannot be dismissed, it will become feasible to target several members of the same miRNA family to analyze the phenotypic consequences, thanks to improvements in mouse genetic manipulation. This strategy has already been achieved in zebrafish [120]. This is especially relevant as the study of alternative animal models can also provide novel insights regarding the role of miRNAs during retinal development, taking advantage of fast life cycles and large progenies, under the assumption that fundamental regulatory mechanisms can be reasonably conserved across vertebrate evolution. Recently constituted as a model for retinal miRNA-based regeneration [121], the retina of zebrafish is also governed by miRNA during development (e.g., miR-9, miR-18, miR-20a, miR-216a) [122,123,124,125].

At the molecular level, omics at the single-cell/nucleus level enables the discovery and characterization of novel retinal cell subtypes ([126,127,128,129,130] among others) and allows the analysis of cellular lineages and different developmental states with unprecedented resolution. To highlight the power of this approach, a recent study reports the conserved gene regulatory networks between mouse and human retina during development using information from single-cell expression (scRNA-seq) and chromatin accessibility (ATAC-seq); this study exemplifies the relevance of the NFIA/B/X factors in the temporal identity of RPCs, and the interplay between INSM1/2, TCF7L1/2, and TBX3 in postnatal rod specification [131]. How these factors regulate miRNAs will add an unprecedented overview of the orchestration underlying the development of the retina and concurrent eye disorders.

## Figures and Tables

**Table 1:** Summary of the most relevant results and experimental evidence regarding miRNAs in mouse retinal development. EC, endothelial cells; ERG, electroretinogram; FACS, fluorescent activated cell sorting; HRMEC, human retinal microvascular endothelial cells; ISH, in situ hybridization; MG, Müller glia; OCT, optical coherence tomography; OE, over-expression; OIR, oxygen-induced retinopathy; PAR-CLIP, photoactivatable ribonucleoside-enhanced crosslinking and immunoprecipitation; RPC, retinal progenitor cells; RPE, retinal pigment epithelium; scRNA-seq, single cell RNA-seq.

miRNA	Evidence	Experimental Approach	Role	Validated Targets	Experimental Target Validation	Ref.
let-7a	Decreased expression in *Dicer1* cKO (*αPax6cre; R26EYFP; Dicer^fl/fl^*)	RT-qPCR	Dicer-dependent miRNA, part of the transition from early to late progenitors (E12-E16)			[38]
	Increased expression during development (E0–P3) in C57BL/6, enriched in progenitor Hes5^+^ cells; use of mimics rescued *Dicer1* cKO phenotype	Microarray, RT-qPCR, plasmid transfection		*Prtg*, *Lin28b*	Antiparallel expression, upregulated in *Dicer1* cKO, luciferase assays, OE maintains early progenitor competence	[39]
let-7c	Increased expression in adult MG (FACS-purified from *Rlbp1-CreER:* *tdTomato^flSTOP/flSTOP^* mice) compared to P2 RPC (FACS-purified from *Sox2-CreER: tdTomato^flSTOP/flSTOP^* mice)	nCounter	Antagonistic role to miR-25 and miR-124 in MG differentiation	Ascl1 (and other mature neuronal markers?)	Increase in the number of Ascl1:tdTomato+ cells, increase in Ascl-reporter activity, defective differentiation after miR-25 mimic/let7a antagomiR (scRNA-seq)	[40]
let-7f	Increased expression during development (E10–P3) in C57BL/6, enriched in progenitors Hes5^+^ cells; use of mimics rescued *Dicer1* cKO phenotype	Microarray, RT-qPCR		*Prtg*, *Lin28b*	Antiparallel expression, upregulated in *Dicer1* cKO, luciferase assays, maintenance of early progenitor competence in target OE assays	[39]
miR-7a	Expression pattern of increase (E14–P0), decrease (P0–P2), and increase (P2–adult) in ICR mice. Suppressed RPC differentiation into MG without perturbing proliferation	RT-qPCR, transfection	Negative MG differentiation	Notch3	Luciferase and IHC assays after miR-7 or Notch3 manipulation levels	[41]
miR-9	Preferentially expressed in retina compared to brain and heart. Increased expression during development (peak at P10) in SVJ129 mice	Microarray, RT-qPCR				[42]
	Decreased expression during development (P4–adult) in C57BL/6J mice	RT-qPCR		ACCN2, ETS1, KLF13, LIN28B, SH2B3	Pool of miR-124, miR-125, and miR-9 in HEK293 cells	[43]
	Decreased expression in *Dicer1* cKO (*αPax6cre; R26EYFP; Dicer^fl/fl^*)	RT-qPCR	Dicer-dependent miRNA, part of the transition from early to late progenitors (E12-E16)			[38]
	Increased expression during development (E10–P3) in C57BL/6, enriched in progenitors Hes5+ cells; use of mimics rescued *Dicer1* cKO phenotype	Microarray, RT-qPCR, transfection		*Prtg*, *Lin28b*	Antiparallel expression, upregulated in *Dicer1* cKO, luciferase assays, OE maintains early progenitor competence	[39]
	Decreased expression from DIV8 to DIV14 in cultured MG	nCounter				[44]
	OE of miR-9/9* and miR-124 suppresses RPC differentiation in glial cells and promotes their differentiation into neurons (P3, P14) in Slc:ICR mice	Plasmid electroporation	Regulation of cell fate in RPC			[45]
	Decreased expression of miR-9-5p during development (P0–P21) in C57BL/6J mice	Microarray, RT-qPCR				[46]
	Preferentially expressed in retina compared to brain and heart. Increased expression of miR-9-3p during development (E10–adult) in SVJ129 mice	Microarray, RT-qPCR				[42]
	Increased expression of miR-9-3p during development (P0–P21) in C57BL/6 mice	Microarray, RT-qPCR				[46]
miR-18a-5p	Decreased expression during development (P1–8w) in C57Bl/6 mice, suppression of endothelial function of HRMEC using agomiR	Small RNA-seq, RT-qPCR	Negative regulator of angiogenesis	FGF1, HIF1A	Luciferase reporter and Western blotting assays after manipulation of miR-18a-5p levels	[47]
miR-21	Increased expression in human-cultured RPE (1 to 4 weeks) and mouse RPE/choroid/sclera explants (4 to 22 months)	RNA-seq of exosomes, RT-qPCR, ISH	Activation of p53 pathway in retinal microglia	*Cdkn1a*, *Cdc25a*, *Daxx*	RT-qPCR in transfected cells with miR-21 mimics	[48]
miR-24	Decreased expression in *Dicer1* cKO (*αPax6cre; R26EYFP; Dicer^fl/fl^*)	RT-qPCR	Dicer-dependent miRNA, part of the transition from early to late progenitors (E12-E16)			[38]
miR-25	Preferentially expressed in retina compared to brain and heart. Increased expression during development (peak at P10) in SVJ129 mice	Microarray, RT-qPCR				[42]
	Decreased expression during development (P4–adult) in C57BL/6J mice					[43]
	Decreased expression in adult MG (FACS-purified from *Rlbp1-CreER:* *tdTomato^flSTOP/flSTOP^* mice) compared to P2 RPC (FACS-purified from *Sox2-CreER: tdTomato^flSTOP/flSTOP^* mice)	nCounter	Neurogenic role: adult MG reprogramming to neuronal/RPC phenotype	Ascl1 (and other mature neuronal markers?)	Increase in the number of Ascl1:tdTomato+ cells, increase in Ascl-reporter activity, defective differentiation after miR-25 mimic/let7a antagomiR (scRNA-seq)	[40]
miR-29a	Increased expression in *Nrl* KO mice	Microarray, RT-qPCR				[49]
	Increased expression during in vitro RPC differentiation	RT-qPCR	Inhibition of RPC proliferation and induction of differentiation	RBM8A	Decreased mRNA and protein expression in RPC differentiation, luciferase assay	[50]
	Decreased expression of miR- 29a-3p during development (P0–P21) in C57BL/6 mice	Microarray, RT-qPCR	Inhibition of retinal angiogenesis. Potential antiangiogenic factor in oxygen-induced retinopathy	SGK3	Protein and mRNA change following manipulation of miR-96a-3p levels	[51]
miR-96	Preferentially expressed in the retina compared to brain and heart. Increased expression during development (E10–adult) in SVJ129 mice	Microarray, RT-qPCR	Regulation of circadian-dependent expression	*Adcy6*	Antiparallel circadian expression and decrease luciferase activity	[42]
	64 bp deletion of miR-96-5p/3p sequences results in developmental delay of photoreceptor cells	Characterization of KO mice: IHC, ERG	Indispensable for the maturation of photoreceptors, especially cones	Several (potentially > 400 genes)	Differential expression mutant vs. wt (P120) in RNA-seq	[52]
miR-124	Increased expression during development (P4–adult) in INL and ONL of C57BL/6 mice	RT-qPCR, ISH		ACCN2, ETS1, KLF13, LIN28B, SH2B3	Pool of miR-124, miR-125, and miR-9 in HEK293 cells	[43]
	Decreased expression in *Dicer1* cKO (*αPax6cre; R26EYFP; Dicer^fl/fl^*)					[38]
	Increased expression of miR-124-3p during development (P0–P21) in C57BL/6J mice	Microarray, RT-qPCR				[46]
	OE of miR-9/9* and miR-124 suppresses RPC differentiation in glial cells and promotes their differentiation into neurons (P3, P14) in Slc:ICR mice	Plasmid electroporation	Regulation of cell fate in RPC			[45]
	KO mice for the precursor of miR-124a, Rncr3/LINC00599, shows abnormal brain including retinal cone cell death	RT-qPCR	Survival of cone photoreceptors	LHX2	Luciferase and IHC assays	[53]
miR-125a	Decreased expression during development (P4–adult) in C57BL/6J mice, enriched at ONL and INL	RT-qPCR, ISH		ACCN2, ETS1, KLF13, LIN28B, SH2B3	Pool of miR-124, miR-125, and miR-9 in HEK293 cells	[43]
	Decreased expression of miR-125a-5p during development (P0–P21) in C57BL/6 mice	Microarray, RT-qPCR				[46]
miR-125b	Increased expression during development (P4–adult) in C57BL/6J mice, enriched at ONL and INL	RT-qPCR, ISH		ACCN2, ETS1, KLF13, LIN28B, SH2B3	Pool of miR-124, miR-125, and miR-9 in HEK293 cells	[43]
	Increased expression during development (E0–P3) in C57BL/6; use of mimics rescued *Dicer1* cKO phenotype	Microarray, RT-qPCR		*Prtg*, *Lin28b*	Antiparallel expression, upregulated in *Dicer1* cKO, luciferase assays, maintenance of early progenitor competence in target OE assays	[39]
	Prominent increased expression of miR-125-5p from P11 to adult MG (FACS-purified from *Rlbp1-CreER:* *tdTomato^flSTOP/flSTOP^* mice)	nCounter				[44]
miR-132	Uniformly expressed in the mouse RGC layer between P0 and P12 and regulated by BDNF in CD-1 mice	IHS	Axonal branching and terminal maturation of RGC	p250GAP (Arhgap32)	Axonal branching effects in OE assays	[54]
miR-143	Decreased expression in *Nrl* KO mice	Microarray, RT-qPCR				[49]
	Increased expression at E16, decreased at P1, and increased at adult C57BL/6 mice	RT-qPCR	Photoreceptor differentiation by Nrl inhibition	Nrl, Neurod1	Luciferase and FACS (GFP) assays and expression analysis after manipulation of miR-143 levels	[55]
miR-145	Increased expression at E16, decreased at P1, and increased at adult C57BL/6 mice	RT-qPCR	Photoreceptor differentiation by Nrl inhibition	Nrl, Crx	Luciferase and FACS (GFP) analysis and expression analysis after manipulation of miR-145 levels	[55]
	Increased expression during development (P7–P17) in C57BL/6 mice	RT-qPCR	Pathological vascularization (OIR model) but little impact on normal capillary development	*Tmod3*	Luciferase assay and decreased levels in EC of OIR model	[56]
miR-155	Manipulation of miR155 levels disrupts physiological angiogenesis (P0–P8) in Lifeact-EGFP wt	Characterization of in vitro matrigel-based assays of microvascular EC	Regulation of neonatal retinal vasculature	SMAD1, SMAD5	Protein expression and phosphorylation change upon manipulation of miR-155 levels	[57]
miR-182	Preferentially expressed in retina compared to brain and heart. Increased expression during development (E10–adult) in SVJ129 mice	Microarray, RT-qPCR	Regulation of circadian-dependent expression	*Adcy6*	Antiparallel circadian expression and decrease luciferase activity	[42]
	Increased expression during development (E13.5–adult) in wt. KO mice had subtle morphological changes, reduced light response, and retinal transcriptional dysregulation	RT-qPCR, characterization of KO mice: OCT, ERG		Several (>100 genes, including *Rho*, *Prph2*, *Pde6b*, *Opn1mw*, *Opn1sw*, *Gnat2*)	Differential expression mutant vs. wt (P7, P42) in RNA-seq and RT-qPCR	[58]
miR-183	Preferentially expressed in adult retina compared to brain and heart. Increased expression during development (E10–adult) in SVJ129 mice	Microarray, RT-qPCR				[42]
	Decreased expression in *Dicer1* cKO (*αPax6cre; R26EYFP; Dicer^fl/fl^*)	RT-qPCR	Dicer-dependent miRNA, part of the transition from early to late progenitors (E12-E16)			[38]
	Increased expression during development (E10–P3) in C57BL/6J mice	RT-qPCR				[39]
	miR-183 KO leads to altered retinal light responses	ERG		*Rnf217*	Decreased expression in retina development and after AAV-miR-183 injection; altered ERG after AAV-Rnf217 injection	[59]
miR-204	Increased expression during development (E15–P12 and adult) in C57BL6 mice; decreased expression in *Nrl* KO adult mice	Microarray, RT-qPCR				[49]
	Increased expression during development (P4–adult) in C57BL/6J mice	RT-qPCR				[43]
	Predominant increase from P11 to adult MG (FACS-purified from *Rlbp1-CreER:tdTomato^flSTOP/flSTOP^* mice) and in cultured MG	nCounter				[44]
miR-211	Preferentially expressed in retina compared to brain and heart. Increased expression during development (E10–adult) in SVJ129 mice	Microarray, RT-qPCR				[42]
	Decreased expression in Nrl KO adult mice	Microarray, RT-qPCR				[49]
	Increased expression during development (E15–P12 and adult) in C57BL6 mice	RT-qPCR				[49]
	miR-211 KO mice lead to a progressive cone dystrophy phenotype with specific cone receptor cell affectation	Immunostaining of cone markers, ERG	Regulation of cone photoreceptor survival and function	Several (>60 genes including *Plin*, *Fbp4*, *Cidec,* and *Pck1*)	Differential expression mutant vs. wt (P7, P42) in RNA-seq	[60]
miR-216a/b-5p	Most downregulated miRNA in PTF1A-defective mice. Decreased expression during development (P0–P12) in CD-1 mice	Small RNA-seq, RT-qPCR, ISH	Amacrine cell formation	*Foxn3*	PAR-CLIP for miR-216a/b sites and luciferase assays after manipulation of miR-216b levels	[61]

## Data Availability

Not applicable.
